# *In Silico* Estimation of Translation Efficiency in Human Cell Lines: Potential Evidence for Widespread Translational Control

**DOI:** 10.1371/journal.pone.0057625

**Published:** 2013-02-27

**Authors:** Stewart G. Stevens, Chris M Brown

**Affiliations:** Biochemistry and Genetics Otago, University of Otago, Dunedin, New Zealand; The John Curtin School of Medical Research, Australia

## Abstract

Recently large scale transcriptome and proteome datasets for human cells have become available. A striking finding from these studies is that the level of an mRNA typically predicts no more than 40% of the abundance of protein. This correlation represents the overall figure for all genes. We present here a bioinformatic analysis of translation efficiency – the rate at which mRNA is translated into protein. We have analysed those human datasets that include genome wide mRNA and protein levels determined in the same study. The analysis comprises five distinct human cell lines that together provide comparable data for 8,170 genes. For each gene we have used levels of mRNA and protein combined with protein stability data from the HeLa cell line to estimate translation efficiency. This was possible for 3,990 genes in one or more cell lines and 1,807 genes in all five cell lines. Interestingly, our analysis and modelling shows that for many genes this estimated translation efficiency has considerable consistency between cell lines. Some deviations from this consistency likely result from the regulation of protein degradation. Others are likely due to known translational control mechanisms. These findings suggest it will be possible to build improved models for the interpretation of mRNA expression data. The results we present here provide a view of translation efficiency for many genes. We provide an online resource allowing the exploration of translation efficiency in genes of interest within different cell lines (http://bioanalysis.otago.ac.nz/TranslationEfficiency).

## Introduction

The nature of a cell, tissue, or organism is largely determined by the precise amounts of specific set of proteins made. Recent transformational advances in molecular technologies have made determining the amounts of mRNA common in many studies. However, to usefully interpret this data we need to understand how mRNA is translated into functional proteins. In the last few years advances in proteomic technologies have made it technically feasible to measure the expression of thousands of proteins, reviewed in [1], [2]. A significant finding from these studies is that there is not a good correlation between the amount of protein and mRNA.

The amount of protein corresponding to the mRNAs for a particular gene depends on how efficiently the mRNAs are translated, translation efficiency (TE) and the protein stability. In a general model of gene expression it is expected that increases in mRNA levels would have concomitant increases in protein, providing that the protein half-life does not vary. Deviations from this simple relationship during changes in gene expression may be due to translational control mechanisms, or could result from variation in translation efficiency of alternative mRNA isoforms [3], [4].

The relationship between mRNA and protein levels has been modelled with differing levels of detail and complexity [4], [5]. A calculation for translation efficiency similar to that used here has been used in previous studies [6], [7]. Alternative measures of estimating TE have been successfully used to model translation, recent examples include ribosome profiling, tRNA Codon Adaptation Indices (tCAI), or other measures of codon bias (e.g. CAI) [8]. Ribosome and polysome profiling have some advantages in that protein data need not be collected [9], [10]. Measures such as CAI and tCAI can be derived directly from the genome but do not allow for much cell specificity [11], [12], these measures have been most useful in single celled eukaryotes and prokaryotes [13].

Proteins mediate some of the best known post-transcriptional regulatory mechanisms – a classic example being the binding to an Iron Responsive Element (IRE) in ferritin mRNAs [14], [15], [16]. Non-coding RNAs such as miRNAs binding to target sites in mRNAs can also effect translation. These can both repress translation and destabilise specific mRNAs, though recent studies have indicated that the predominant form of regulation may be mRNA destabilisation [17]. Modulation of RNA stability is not considered in this study as experimentally determined absolute mRNA levels are used.

To measure gene expression it is presently technically easier to detect mRNA, rather than protein, or indeed functional protein. Therefore, despite indications of widespread translational control mechanisms, many studies utilise mRNA expression as a proxy for gene expression.

Several recent studies have generated large datasets that contain both protein and mRNA levels for thousands of genes [7], [18], [19]. In each study protein levels were determined by mass spectrometry and mRNA levels were determined by high throughput sequencing. Protein stability data were determined using the pulsed SILAC method [20], [21] in the HeLa cell line [22]. These combined datasets have provided the opportunity to compare TE values across different cell lines for many individual genes. This study presents data for 3,990 genes in five human cell lines. It provides a gene by gene comparison of TE and suggests avenues for further research.

## Results

### Messenger RNA and Protein Levels in Five Human Cell Lines

Paired protein and mRNA data for five human cell lines from published studies were available. The MCF-7 and HeLa are the well established breast and cervical cancer cell lines. A-431 is an epidermoid (squamous cell) carcinoma cell line. U-2 OS is an osteosarcoma cell line. U-251 MG is a glioblastoma cell line. The protein and mRNA data were determined by similar methodologies in three different laboratories. The number of paired detectable proteins and mRNA values was 24,920. In total there were paired data for 8,170 unique genes in one or more cell lines. The well studied cervical cancer cells (HeLa) had the most comprehensive dataset with 7,297 pairs. This may indicate a greater sensitivity of protein detection in that study. For 2,156 mRNA and protein pairs, there were data for all five cell lines, and protein stability data was available for 1,807 of these. The data and analysis for each of 8,375 genes and the major groups defined below are available at bioanalysis.otago.ac.nz/TranslationEfficiency and in a supplementary spreadsheet (File S1).

**Figure 1:**
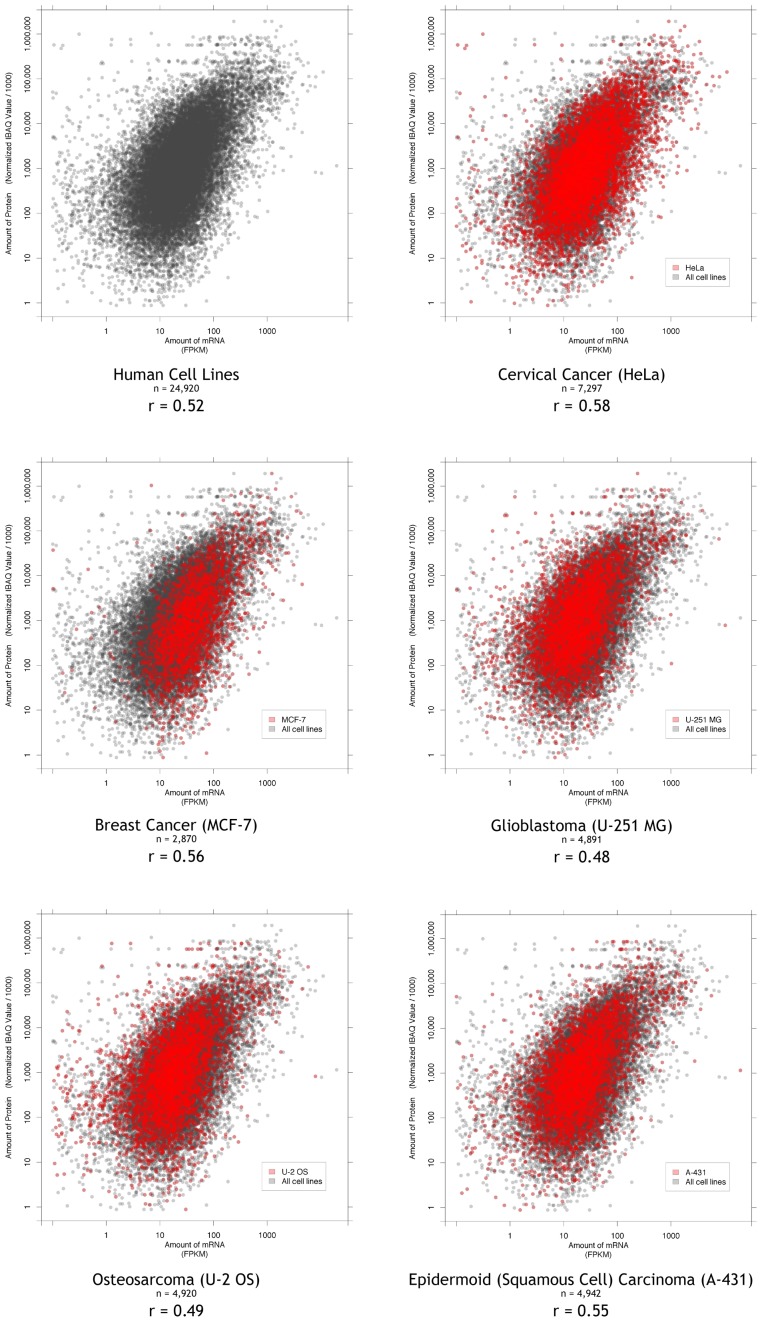
The relationship between mRNA and protein levels for five human cell lines. The source datasets originate from the referenced studies and are processed as displayed to allow comparison (see Methods). Each point on the graphs represents a gene, the amount of mRNA on the x-axis and the amount of protein on the y-axis. The mRNA levels are the counts of Fragments Per Kilobase of exon model per Million mapped reads (FPKM). The protein quantities are Limma normalised intensity based absolute quantification (IBAQ). The first panel shows the combined results and subsequent panels particular human cell lines highlighted in red. The r-values indicate the Spearman’s rank correlation coefficient, n - indicates the number of mRNA/protein pairs for each cell line or in total for the first panel.

In order to compare the five sets of data a common analysis pipeline was used. The amounts of mRNAs (FPKM) and proteins (normalised IBAQ) are plotted on a log scale for all of the data points (24,920 pairs) and for each of the cell lines in Figure 1. The interdecile range of mRNA varies over 2 orders of magnitude, and the protein over 4 orders of magnitude. This demonstrates the ability of these newly developed techniques to capture a wide dynamic range of both protein and mRNA levels. The dynamic range of the protein levels is greater than that of the mRNA, confirming suggestions of the importance of post-transcriptional control from other studies [7].

There is no significant stratification in the data that might indicate technical limitations with high or low abundances, or lack of sensitivity in any of the studies or cells. However, there is some evidence of saturation in the upper protein amounts in the three cells lines (Figure 1, upper red points on last three panels) from the Lundberg study, possibly reflecting a small systematic saturation effect for the abundant proteins.

There are a range of correlations between the amount of protein and the mRNA that encodes it in each cell line (Spearman’s correlation coefficient r = 0.48–0.58). For all the data combined the overall correlation is r = 0.52 and the coefficient of determination, R-squared, R^2^ = 0.28 (Methods). This indicates that assuming a linear model, 28% of the amount of protein can be estimated from the amount of mRNA. The individual cell lines differed but all show a similar distribution of points within the overall dataset (Figure 1). The best correlation, r = 0.58 was seen for the largest dataset – the HeLa cells, in agreement with the previously reported correlation (0.6) from this data prior to processing though our pipeline [19].

These figures represent the overall correlation between protein and mRNA levels. However, some genes show better correlations for the five cells. This is due to either differing mRNA amounts in each cell line with proportional changes in protein levels, or similar amounts of both mRNA and protein in all cell lines.

### Calculation of Estimated Translation Efficiency (TE)

**Figure 2:**
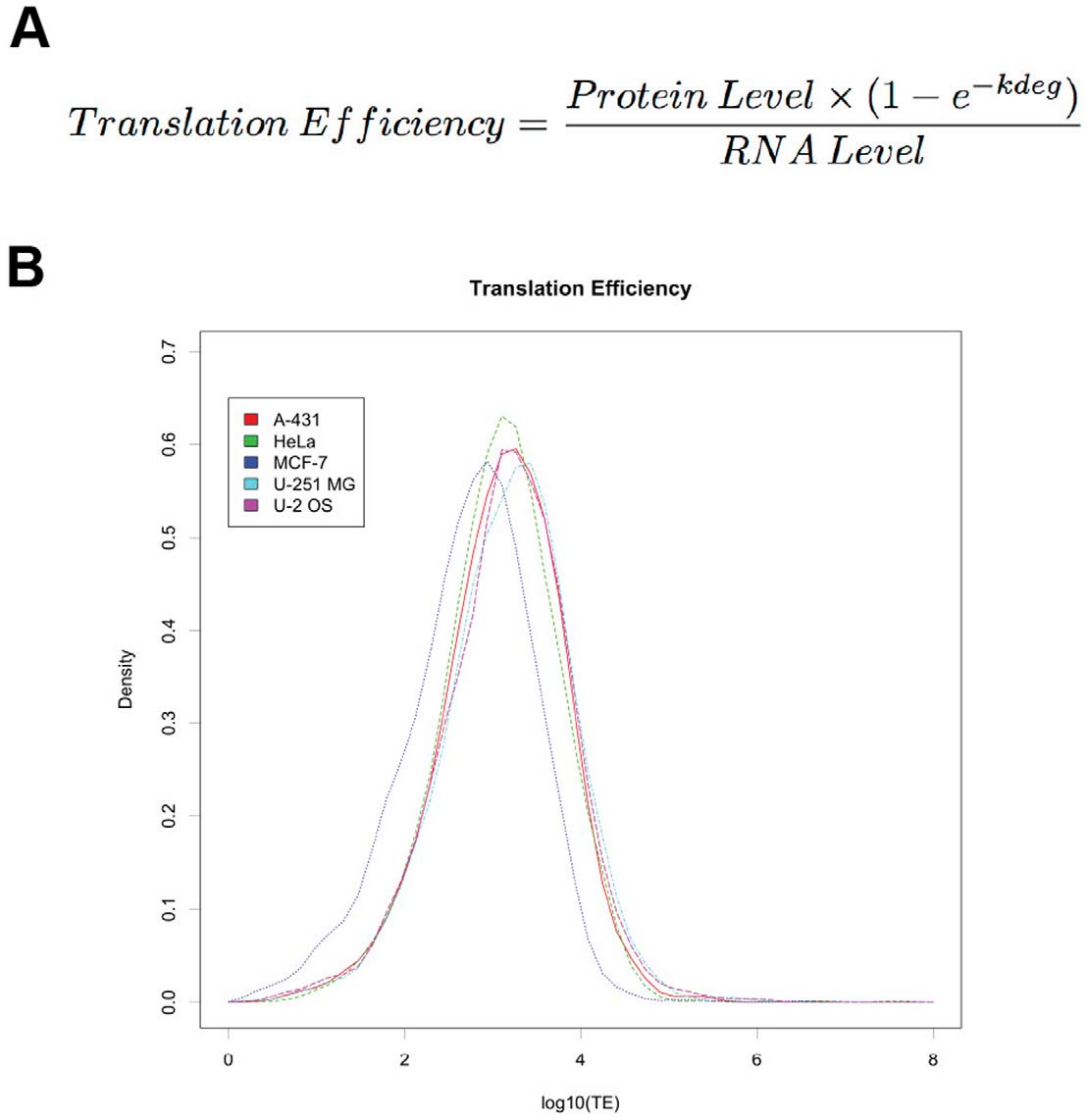
A. Calculation of translation efficiency. This calculation provides a measure of translation efficiency, an important determinant of gene expression. The term that accounts for the protein stability is (1−e^−kdeg^), the protein stability accounting term (PSAT). The *k_deg_* is the decay constant of the protein from Nagaraj 2012. The interquartile range of overall TE is 10 fold and for the PSAT factor 1.85 fold. **B. Distribution of estimated translation efficiency in the five types of cell.** The distributions of TEs are shown for each cell line. The median log_10_TE is 3.12 and SD 0.72 for all cell lines (n = 15,918); A-431 (median: 3.15, SD: 0.69, n = 3,376); HeLa (median: 3.11, SD: 0.67, n = 3,661); MCF-7 (median: 2.79, SD: 0.74, n = 2,158); U 251-MG (median: 3.23, SD: 0.72 n = 3,358); U-2 OS (median: 3.20, SD: 0.73, n = 3,365).

For each gene a relative measure of estimated TE was calculated (formula Figure 2A, Methods and [7]). This calculation includes mRNA levels, protein levels, and protein stability. This calculation also assumes steady state of both mRNA levels and protein levels. The dataset for protein stability used was determined by pulsed SILAC in HeLa cells [22] – this dataset came from the same group as the HeLa mRNA and protein levels. These protein stability values were also used for the other cell lines.

To investigate the effect of applying the protein stability levels from HeLa to other human cell lines, protein stability data collected using similar methods in mouse (NIH-3T3) cell lines [7] were compared for orthologus genes (Figure S1). This result is similar to a comparison between the HeLa cell line and the mouse C2C12 cell line [22]. There was a good protein by protein correlation (r = 0.58), however the mean protein stability determined for the mouse NIH-3T3 cell line is about twice that for the HeLa cell line (half life of ∼40 h vs. ∼80 h). As protein stability is regulated in specific cells, the use of HeLa data is a limitation of our model for non-HeLa cell lines.

The normalised protein stability values and comparison for the NIH-3T3 and HeLa cell lines are available in the material S1.

The term in the TE formula (Figure 2A) which accounts for the protein stability is (1−e^−kdeg^), the protein stability accounting term (PSAT). The interquartile range of this term is 0.015 to 0.027 (1.85 fold) with a median of 0.019. This term scales the ratio of protein level to mRNA level, by accounting for protein stability.

The calculated TE values have the approximately log-normal distribution shown in Figure 2B. The log_10_ (TE) values have a median of 3.12, standard deviation of 0.72. Each of the cell lines had a similar range of TE (Figure 2B).

### The Relationship between Translation Efficiency and Gene Function

**Figure 3:**
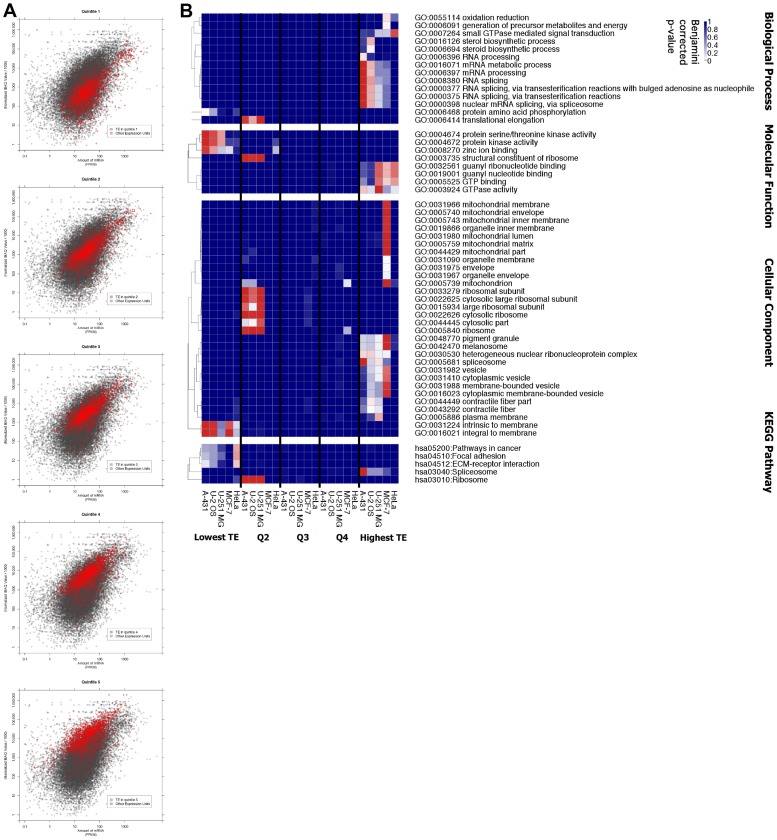
Functional characteristics of genes grouped by estimated translation efficiency. **A.**Quintiles using the median estimated TE for genes with available data in all five cell lines are highlighted in red. Genes with higher TE tend to have higher accumulated protein levels. There are some genes in the combined data (grey) that are not included - these are only expressed in some of the cell lines, or there were no protein stability data available. **B.** Analysis of enrichment within gene ontology (GO) and KEGG pathway classifications for genes within each quintile of TE for each cell line. All ontologies shown have at least one enrichment passing a Benjamini corrected p-value<0.05. The colours in the figure correspond to corrected p-values such that colours from white to red show significance up to a significant p-value<0.05 (red). A spreadsheet of these p-values is included in the File S2.

Genes involved in particular processes, functions or cellular components possibly have similar TE. We tested this idea by dividing the genes present in all five cell lines into five groups by TE (Figure 3A, red points). Each group was analysed for enrichment in Gene Ontology (GO) and KEGG pathway terms (Methods).

Surprisingly, there were no ontologies or pathways that showed significant enrichment over all five cell lines. However, significant (p<0.05, Benjamini corrected) enrichment was observed for individual cell lines, these terms are shown in Figure 3B. The different types of cells showed various enrichments in high or low TE. “GTP binding” showed enrichment within the high TE genes for the HeLa, MCF-7 and U-251 MG cell lines. The biological process, “small GTPase mediated signal transduction” shows enrichment for high TE in HeLa cells. Notably, mitochondrial groups show enrichment within the high TE genes in MCF-7 cells. The A-431 and U-2 OS cell lines also had significant enrichment for “RNA splicing” within the high TE genes. The U-2 OS cell line showed enrichment for “steroid biosynthetic process” within high TE genes. There was significant enrichment for KEGG “pathways in cancer”, “focal adhesion” and “ECM-receptor interaction” within the low TE genes for the HeLa cell line. Interestingly within the second lowest TE quintile the A-431, U-2 OS and U-251 MG cell lines all show enrichment for the ribosome cellular component and KEGG pathway in addition to several similar terms (Figure 3B).

In order to assess the robustness of the estimated TE data and enrichments, a similar analysis using NIH-3T3 protein stability data was completed. Detailed results are shown in Figures S2 and S3.

### The Relationship between Translation Efficiency and Protein Stability

**Figure 4:**
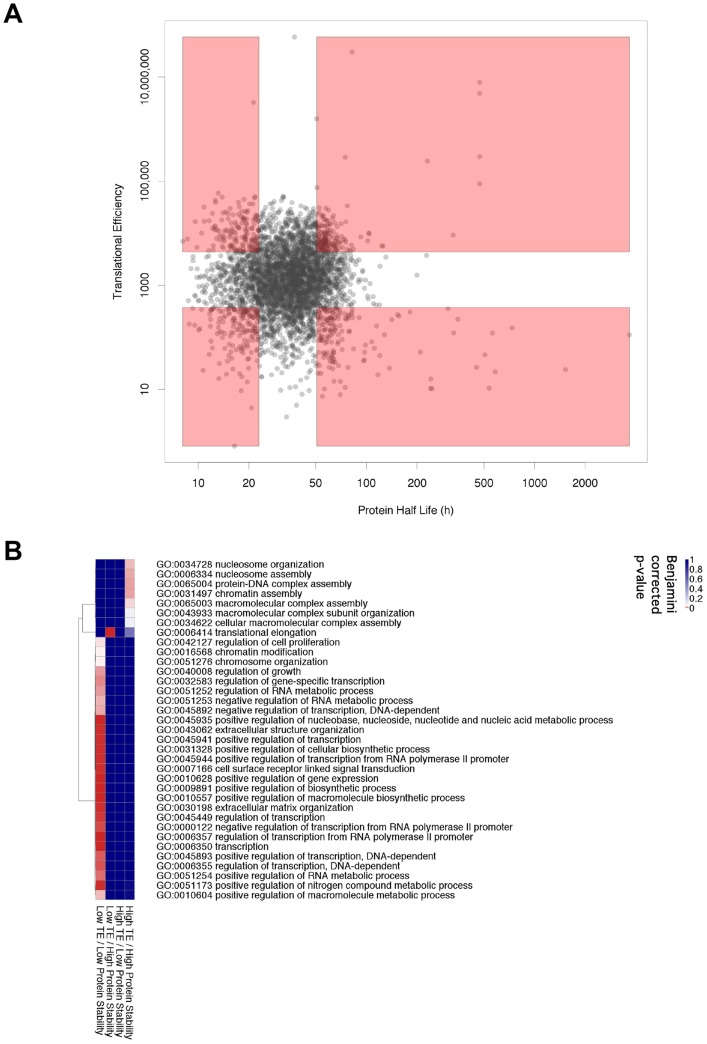
Relationship between translation efficiency and protein half life in HeLa cells. **A.** Plot of the translation efficiency versus the protein half life for each gene expressed in HeLa cells. The pink highlighting indicates intersections between the upper and lower quintiles. **B.** Enrichment of gene ontology classes for the four groups of genes highlighted in 4A. All classes shown have at least one enrichment passing a Benjamini corrected p-value<0.05. A spreadsheet of these p-values is included in the File S2.

TE and protein stability might be correlated, as for example, genes with a high TE might have high protein stability, both increasing protein levels. We tested for correlation in the HeLa cell data. For the HeLa cells the relationship between TE and half life of the protein is shown in Figure 4A. The inter quintile range for TE is ten fold, but for protein half life it is less than two fold. Surprisingly, there is no significant overall correlation between TE and protein stability (Figure 4A), despite protein stability being included in the TE calculation.

It might be expected that TE and protein stability would work synergistically to give high or low expression for classes of genes. Therefore sets of high TE + high stability (Figure 4A, upper right, quintiles), and low TE + low stability (Figure 4A, lower left, quintiles) might be expected to show enrichment for specific classes of genes. We have analysed these two expected groups, and the other two combinations, more closely. Figure 4B shows those terms with significant enrichment in intersecting quintile groups.

The genes that have both low TE and low protein stability are significantly enriched for those involved in “regulation of growth”, “regulation of transcription”, “chromatin modification” and “extracellular matrix organization”. An example in this class of genes is the STAT6 transcription factor which has a protein half life of 18 hours, PSAT = 0.038 and a log_10_ TE of 2.57. The IBAQ value of 555,142 is in the 2^nd^ quartile for HeLa cells and the FPKM value of 56 is in the 3^rd^ quartile for HeLa cells.

The genes that have high TE and high protein stability are significantly enriched for those involved in “nucleosome assembly” and “macromolecular complex assembly”. These include mainly histones and other DNA binding proteins. The histone mRNAs lack polyA tails and so their transcripts may be underrepresented. An example of this class of gene (other than a histone) is TUBB3– from the tubulin protein family. The TUBB3 gene has a protein half life of 65 hours, PSAT = 0.011 and a log_10_ TE of 3.68. The IBAQ value of 526,639 is in the 2^nd^ quartile for HeLa cells and the FPKM value of 1 is in the 1^st^ quartile for HeLa cells.

The two groups where TE and protein stability go in opposing directions show enrichment in only one term. Genes with a low TE but high protein stability are enriched for the GO term, “translational elongation”. These include 15 very stable ribosomal proteins and a translation elongation factor. The median log_10_ TE for these ribosomal proteins is 2.18 and the median protein half life is 72 hours (PSAT = 0.0096).

A spreadsheet of the classes and p-values, also including those not passing the stringent Benjamini correction, is included in the File S2.

### Variation of Estimated Translation Efficiency for Genes in Different Cell Lines

**Figure 5:**
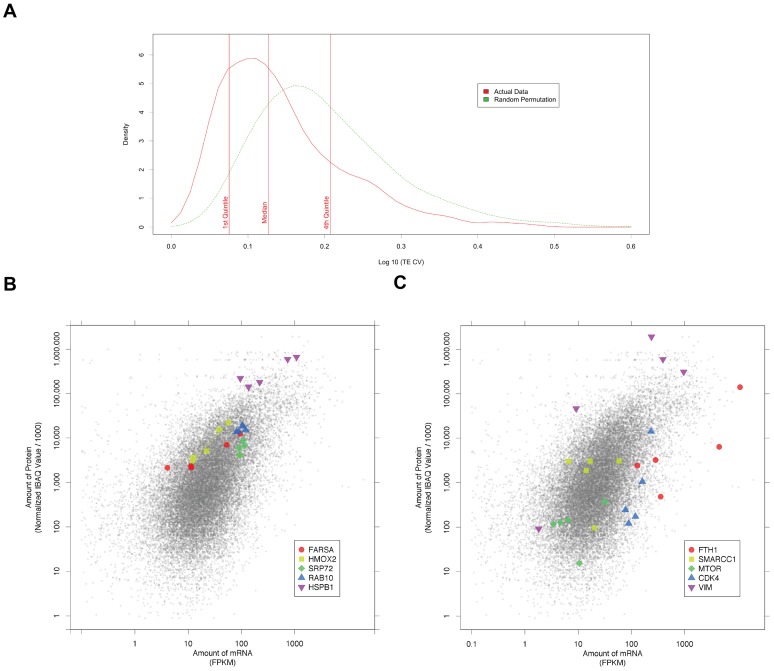
Grouping of genes by variation in estimated translation efficiency. **A.**The distribution for the coefficient of variation (CV) of the estimated TE for all genes is shown with the quintiles marked. A similarly calculated distribution using randomly generated TE values is shown for comparison. The real data has a lower TE CV than would be expected randomly. **B. Genes with a small variation in TE.** Some examples of well-studied genes are shown. They either have similar expression values at both the protein and mRNA level (e.g. RAB10, SRP72) or have linear relationships between these values (e.g. HMOX2, HSPB1, FARSA). A line is drawn representing this latter relationship where the p-value is<0.01. **C. Genes with a large variation in TE.** Estimated translation efficiency for some genes is highly variable between cell lines. Examples indicated here are FTH1, which has well studied translational control mechanisms. Some genes (e.g. SMARCC1) have large variation in the amount of mRNA but not in the amount of protein. Conversely other genes (e.g. CDK4) have a large variation in protein but little variation in mRNA. Other genes (e.g. VIM) vary in both protein and mRNA amounts.

In order to determine if specific genes had similar TEs in the different cell lines, we calculated the coefficient of variation for the TE (TE CV, the standard deviation divided by the mean, Methods). This was done for the 1,807 genes with a TE value in each cell line. The distribution of TE CV across the datasets is shown in Figure 5A. Some genes have very small variations in TE, and therefore have CVs near 0. This is postulated to have a biological basis, however it is possible that many genes may have similar TEs by chance. To assess this, we permuted the genes and TE values 20 times and determined the spread of the TE CV. The random sets had significantly higher variation in TE indicating selection for consistent TE in different cell lines for some genes (t-test p-value<2.2×10^−16^).

Genes with low or high variation in TE may be enriched in particular categories. To address this the data was first divided into five groups of 362 genes by ranking on TE CV. Protein level is closely coupled to the mRNA levels in the 20% with the lowest TE CV (those with a log_10_ TE CV of less than 0.075, Figure 5A). Notably only 4% of randomly permuted TEs were so consistent (Figure 5A). This low TE CV group are enriched in genes involved in the molecular function gene ontology class, “RNA binding”.

The data for five individual genes with low variation in TE are shown in Figure 5B. These genes have been shown as they were well studied and show representative ranges of expression. More detailed data for each of the 1,807 genes is available on the companion website. The most extreme range in expression among genes in the lowest quintile of TE CV was for FARSA (phenylalanyl-tRNA synthetase, alpha subunit) – this showed a low variation in estimated translation efficiency over a 23 fold change in mRNA level between different cell lines.

For the RAS member (RAB10) expression of both mRNA and protein is high in all the cell lines (blue triangles), but there is little difference between the points (log_10_ TE = 3.55±0.07, protein half life 32 h, PSAT = 0.021). In contrast for the heme oxygenase (HMOX2), there are cell type specific changes in expression. There are a five fold range of mRNA levels in the five cell lines (yellow squares) however protein levels are proportional (log_10_ TE = 3.77±0.1, protein half life 35 h, PSAT = 0.020) and a line is shown to indicate that a log-linear model closely describes this gene's data (R^2^, 0.95, p = 0.003). SRP72 and HSPB1 also show consistent TEs (log_10_ TE = 2.96±0.1, 4.11±0.2). They have similar protein half lives (44 h, 43 h, PSAT = 0.016) this indicates that HSPB1 is translated with a consistently (∼14 fold) higher efficiency than SRP72.

In contrast, the data for selected genes with a large variation in TE (quintile with TE CV >0.21) are shown in Figure 5C. The genes shown represent genes that have known translational control mechanisms (FTH1, VIM) and other well studied genes (SMARCC1, CDK4, MTOR). Genes such as SMARCC1 have large variation in the amount of mRNA with smaller variation in the amount of protein. Conversely genes such as CDK4 have a wide range of protein levels but little difference in the amounts of mRNA. Other genes such as VIM vary differently in both protein and mRNA amounts.

## Discussion

In this work data from several different studies have been integrated and reanalysed to gain insight into potential translational control mechanisms in human cells. In the five cell lines analysed there is limited overall correlation between mRNA levels and protein. Messenger RNA levels predict 24–34% (R^2^ values) of individual protein levels using log-normal models, this is consistent with correlations from prior studies [1], [7]. Non-linear models might improve this prediction, although in other studies using such models similar relationships were obtained (27%) [23].

The primary protein and mRNA data were collected by three different groups. These datasets were processed in this study using a standard pipeline. This pipeline aimed to minimise differences in read lengths and mapping strategies between the original studies. The ranges of values for FPKM and IBAQ values were similar in the cell lines (Figure 1, 2). As more datasets become available these can be integrated into our analysis system.

### Translation Efficiency

We have used a previously described calculation for TE that utilises mRNA levels, protein levels and protein stability (Figure 2A). TE has previously been defined as the number of protein molecules translated from an mRNA per unit time (proteins per mRNA per h) and this is an intuitively useful unit [7]. For a published mouse cell line, where absolute quantification was possible due to inclusion of an internal standard (human) the median was 43.6 proteins per mRNA per h and interquartile range 17–93. Without calibration against known values or internal standards the TE calculated in this study has relative units. If overall TEs in human and mouse cell lines are similar then the TE median of 1,318 (log_10_ (TE) 3.12, Figure 2B) would correspond to 43.6 proteins per mRNA per h, and the interquartile range of these human TEs 444–3,648 corresponds to 17–93 proteins per mRNA per h.

Translation efficiencies varied widely for individual genes (Figure 3). Although, some classes of genes were highly translated in some cell lines (e.g. “spliceosome” genes in A-431 cells), grouping by median TE showed no significant enrichments within GO groups or KEGG pathways. For some abundant proteins the TE was not high. For example, for the 98 genes encoding the “cytosolic ribosome” both proteins and mRNAs were abundant, however the mean protein half was high at 68 h, PSAT = 0.010 (HeLa) thus the median log_10_ (TE) was low (2.93– in the second quintile and 3.02 in HeLa cells).

The inclusion of protein stability is a significant difference between the TE calculation used here and a simple protein/mRNA ratio. If protein stability were not taken into account then the “cytosolic ribosome” mRNAs would fall into the third quartile of a simple ratio calculation. Ribosomal mRNAs are translationally regulated by the TOP element - a sequence of pyrimidines at the 5′ terminal. Messenger RNAs with TOP elements are translationally repressed in slow growth. In growing cells about 30% of these mRNAs are sequestered and not translationally active [24]. In resting cells this percentage is higher. The analysis of TE presented here is an average for all mRNAs in the cell and for all cells. A particular gene may have some mRNAs that will be translated more or less efficiently and cells will be in various states of growth. If the fraction of ribosomal mRNAs that are sequestered and translationally inactive is estimated at 30% it would mean that the log_10_ (TE) for the “cytosolic ribosome” mRNAs would be 3.08– close to the median. Other studies also indicate that proteins involved in translation are regulated translationally [10].

The analysis presented in this study provides a large set of genes and estimated TEs that may be further investigated to identify transcript features common to high or low TE. Previous studies have used transcript, coding sequence or UTR characteristics such as length, predicted structure and the presence of upstream open reading frames (uORFs) to help build models capable of predicting protein levels from RNA levels [4], [8], [23], [25]. Directly measured or estimated TE offers the possibility of building improved models for the prediction of protein levels from mRNA levels.

### Protein Stability

Protein stability data is required to accurately estimate TE (Figure 2). In this study protein stability data from the HeLa cell line was used [22]. This was published by the same group as the expression data for this cell line [19]. We therefore expect the TE values will be most accurate for HeLa cells. The experimentally determined stability data was used as an estimate of protein stability in the other human cell lines, although tissue or cell specific regulation of protein turnover is important [26]. Our model could be improved by the use of further protein stability datasets as these become available.

Previous studies have used computational estimates of protein stability predicted from sequences of the proteins [23], this also generates a single cell type independent value. These stability prediction methods are still under active development and have not yet been tested, improved or refined by the extensive protein stability data used here [27], [28].

TE values have a wider range than protein stability values, furthermore we found no overall correlation between TE and protein stability (Figure 4). This supports previous studies that showed that protein stability lies within a narrower range than mRNA or protein abundance [1]. Previous findings also showed protein stability to be a relatively small contributor to overall gene expression in mouse (NIH-3T3) cells [7] and for some *S. cerevisiae* genes [29] although this may be a larger contributor in bacteria [30].

### Variation in Translation Efficiency

This study shows that there was much less variation in TE across cell lines than expected by chance (Figure 5A). This analysis of variation is equivalent to determining the coefficient of variation for the mRNA/protein ratio as the estimate of protein stability used here is the same for every cell line. Some genes show not only low TE variation but also similar expression of the message and protein in all cell lines. These could be useful as internal mRNA or protein level controls across cell lines. As selection of internal controls is critical for quantitative comparisons across tissues there has been much gene-by-gene analysis done of candidate controls [31], [32], [33], [34].

For some genes with low variation in TE between cell lines there can still be proportional differences in protein levels and mRNA levels. For 88 genes of the 362 genes with the smallest TE variation there is over a five fold range in mRNA levels. For 114 genes of these 362 genes with smallest TE variation there is over a five fold range in protein levels. These genes provide good targets for studying translation without cell specific control.

Analysing gene expression data at the mRNA level has been a challenge for understanding biological function and the elucidation of many diseases. Often high throughput results present many targets for follow up. Changes in mRNA levels for genes with low TE variation would be more likely to result in a change at the level of protein and a biological effect. The degree of variation seen in translation efficiency could be incorporated into tools that rank gene candidates [35].

### Possible Examples of Translational Control

Many genes exhibit a large variation in TE between different cell lines (362 genes in the upper quintile, Figure 5A, 5C). These large variations (CV >0.21) though partly due to noise in the underlying data (about 20%, Figure 5A), reflect underlying differences in biological processes in these cells. In particular this pattern of varying efficiencies between different cell lines would be consistent with different translational control mechanisms acting in these cells. This could be protein or RNA (e.g. miRNA) mediated. As examples in support of this idea, two genes (FTH1, VIM) with well established cell or environment specific translational control mechanisms have significant TE variation. For the ferritin heavy chain 1 mRNA (FTH1) there is an IRE in the 5′ UTR that inhibits translation depending on iron levels [14]. Subtle differences in iron in the media between the studies or in iron/oxygen metabolism between the cell lines could produce significantly different TEs [36]. The vimentin (VIM) message is localised within some cells and such localisation is often coupled with translational control [37].

In this study we could not separate the mRNAs or proteins corresponding to alternatively spliced transcripts from the same gene. Alternatively spliced transcripts may be translated with different efficiencies, particularly when these alter UTRs [3], [16], [29]. Therefore, differences in translation efficiency identified in this study may be explained by differential expression of splice variants, differences in protein stability and/or by active translational control mechanisms.

This study has analysed the relationship between protein and mRNA levels in human cell line data. Large scale quantitative data is becoming available for more complex systems, e.g. plants or animals [38], [39], [40] and the methodology described here will be applicable to new datasets as they become available. Comparing data from non-human species would reveal conservation and differences in the regulation of gene expression [41].

We provide an analysis in a graphical form for each of 8,170 genes on a companion website. Researchers can examine the data for their own gene of interest or groups of interesting genes. We also provide all the processed data for additional bioinformatic analysis.

## Materials and Methods

Figure S4 shows a summary of the pipeline used to estimate translation efficiency in the five cell lines.

### Data

Published NGS and IBAQ data were obtained for MCF-7 and HeLa cell lines [7], [19]. For the A-431, U-2 OS and U-251 MG cell lines [18] published NGS data were also available and the proteomic data were available as mass spectrometry intensities. The MCF-7 and HeLa are the well established breast and cervical cancer cell lines. A-431 is an epidermoid (squamous cell) carcinoma cell line. U-2 OS is an osteosarcoma cell line. U-251 MG is a glioblastoma cell line.

The transcript data for the MCF-7 cell line [7] were acquired from 36 base reads and the HeLa cell line [19] were acquired from 76 base reads both using the Illumina GAIIx platform. Transcript data for the A-431, U-2 OS and U-251 MG cell lines [18] were acquired from 50 base reads using the SOLID sequencing platform. The reads from all studies were trimmed to 36 bases and the tophat/cufflinks pipeline [42], [43] was used to map these and compute FPKMs.

For A-431, U-2 OS and U-251 MG the intensity data was converted to IBAQ values using the method previously followed for the MCF-7 cell line [7] (see below). For MCF-7 and HeLa the published IBAQ values were used.

### Conversion of Intensity Data to IBAQ Values

Initially the sophisticated model of PeptideCutter from ExPASy [44] was used to predict trypsin peptide fragments for each protein. A count was made of all peptides in this prediction between 6 and 30 amino acids in length. The intensity values from the Lundberg dataset were divided by these peptide counts to give IBAQ values. A small proportion of the data (47/5237) related to proteins with a varying number of predicted peptides - these were excluded from the analysis.

### Data Pre-processing

The FPKM (Fragments Per Kilobase of exon model per Million mapped reads) data counts are intrinsically a normalised dataset. The IBAQ values were normalised to ensure the average intensities had the same empirical distribution between the different cell lines studied. The limma package from bioconductor [45] was employed for this purpose – using the normalizeBetweenArrays function with the “Aquantile” method. Data points with an FPKM value less than 0.1 were excluded from analysis. The FPKM, IBAQ, TE and Protein Stability (kdeg) values are available in File S1.

### Estimated Translation Efficiency (TE)

An estimated measure of translation efficiency was calculated by using three experimental values: the amount of protein, the amount of mRNA, and protein stability. This is shown in the formula in Figure 2A. At the time the determination is made the amount of each protein and mRNA is assumed to be in a steady state. This means that the amount of newly synthesized protein is equal to the amount of protein being degraded.

Protein stability data was obtained from pulsed SILAC experiments on HeLa cells. In absence of more cell specific information, the protein stability data from the HeLa cell line [22] were used as an estimate of stability in the other human cell lines. In support of this use, there was a strong correlation between protein stability in HeLa and both NIH-3T3 (Figure S1) and C2C12 mouse myoblast [22] cell lines.

The estimated translation efficiency (TE) was calculated for those genes where protein stability data were available. Where data were available for all five of the human cell lines, the median and coefficient of variation (CV) of the translation efficiency was calculated on a gene by gene basis.

### Gene Ontology Analysis

The genes were divided into quintiles based on their median and per cell line estimated translation efficiency. These were uploaded to DAVID [46] together with a background consisting of all the genes for which cell line/median translation efficiency data were available. The Functional Annotation Charts denoted by “GOTERM_BP_FAT”, “GOTERM_BP_FAT”, “GOTERM_BP_FAT” and “KEGG_PATHWAY” were used – these are ontologies which have had the broadest terms filtered. Thresholds were changed to a gene count of 2 and EASE score of 1 (modified Fisher exact p-value). Ontologies were filtered to include enrichments with Benjamini corrected p-values<0.05.

In a similar analysis to the above, the genes in the upper and lower quintiles of translation efficiency in HeLa cells were intersected with those in the upper and lower quintiles of protein stability in HeLa cells. A background was used of all the genes where both protein stability and translation efficiency data were available.

## Supporting Information

Figure S1 **Comparison of protein stability for orthologous genes in HeLa and NIH-3T3 cells.**
(TIF)

Figure S2 **Functional characteristics of genes grouped by estimated translation efficiency calculated from HeLa protein stability data.** Analysis of enrichment within gene ontology (GO) and KEGG pathway classifications for genes within each quintile of TE for each cell line. All ontologies shown have at least one enrichment passing a Benjamini corrected p-value<0.05. The colours in the figure correspond to corrected p-values such that colours from white to red show significance up to a significant p-value<0.05 (red). Only genes with protein stability data available in HeLa and NIH-3T3 cells are considered in the analysis.
(TIF)

Figure S3 **Functional characteristics of genes grouped by estimated translation efficiency calculated from NIH-3T3 protein stability data.** Analysis of enrichment within gene ontology (GO) and KEGG pathway classifications for genes within each quintile of TE for each cell line. All ontologies shown have at least one enrichment passing a Benjamini corrected p-value<0.05. The colours in the figure correspond to corrected p-values such that colours from white to red show significance up to a significant p-value<0.05 (red). Only genes with protein stability data available in HeLa and NIH-3T3 cells are considered in the analysis.
(TIF)

Figure S4 **The pipeline used to estimate translation efficiency in the five cell lines.**
(TIF)

File S1 **The FPKM, IBAQ, TE and Protein Stability data are provided in this spreadsheet. This data is also available on the companion website (** http://bioanalysis.otago.ac.nz/TranslationEfficiency).
(XLS)

File S2 **A spreadsheet of the numeric results from the enrichment analysis, including those terms not passing Benjamini correction.** Only those passing this filter are shown in the paper.
(XLS)

Material S1 (DOC)

## References

[1] Vogel C, Marcotte EM (2012) Insights into the regulation of protein abundance from proteomic and transcriptomic analyses. Nat Rev Genet 13: 227–232. 22411467 10.1038/nrg3185 PMC3654667

[2] Geiger T, Wehner A, Schaab C, Cox J, Mann M (2012) Comparative proteomic analysis of eleven common cell lines reveals ubiquitous but varying expression of most proteins. Mol Cell Proteomics 11: M111 014050. 10.1074/mcp.M111.014050 PMC3316730 22278370

[3] Moore MJ, Proudfoot NJ (2009) Pre-mRNA processing reaches back to transcription and ahead to translation. Cell 136: 688–700. 19239889 10.1016/j.cell.2009.02.001

[4] Gingold H, Pilpel Y (2011) Determinants of translation efficiency and accuracy. Mol Syst Biol 7: 481. 21487400 10.1038/msb.2011.14 PMC3101949

[5] Amman F, Flamm C, Hofacker I (2012) Modelling Translation Initiation under the Influence of sRNA. Int J Mol Sci 13: 16223–16240. 23203192 10.3390/ijms131216223 PMC3546686

[6] Hargrove JL, Schmidt FH (1989) The role of mRNA and protein stability in gene expression. Faseb J 3: 2360–2370. 2676679 10.1096/fasebj.3.12.2676679

[7] Schwanhausser B, Busse D, Li N, Dittmar G, Schuchhardt J, et al (2011) Global quantification of mammalian gene expression control. Nature 473: 337–342. 21593866 10.1038/nature10098

[8] Tuller T, Veksler-Lublinsky I, Gazit N, Kupiec M, Ruppin E, et al (2011) Composite effects of gene determinants on the translation speed and density of ribosomes. Genome Biol 12: R110. 22050731 10.1186/gb-2011-12-11-r110 PMC3334596

[9] Ingolia NT, Lareau LF, Weissman JS (2011) Ribosome profiling of mouse embryonic stem cells reveals the complexity and dynamics of mammalian proteomes. Cell 147: 789–802. 22056041 10.1016/j.cell.2011.10.002 PMC3225288

[10] Tebaldi T, Re A, Viero G, Pegoretti I, Passerini A, et al (2012) Widespread uncoupling between transcriptome and translatome variations after a stimulus in mammalian cells. BMC Genomics 13: 220. 22672192 10.1186/1471-2164-13-220 PMC3441405

[11] Waldman YY, Tuller T, Shlomi T, Sharan R, Ruppin E (2010) Translation efficiency in humans: tissue specificity, global optimization and differences between developmental stages. Nucleic Acids Res 38: 2964–2974. 20097653 10.1093/nar/gkq009 PMC2875035

[12] Mahlab S, Tuller T, Linial M (2012) Conservation of the relative tRNA composition in healthy and cancerous tissues. RNA. 10.1261/rna.030775.111 PMC3312552 22357911

[13] Tuller T, Carmi A, Vestsigian K, Navon S, Dorfan Y, et al (2010) An Evolutionarily Conserved Mechanism for Controlling the Efficiency of Protein Translation. Cell 141: 344–354. 20403328 10.1016/j.cell.2010.03.031

[14] Stevens SG, Gardner PP, Brown C (2011) Two covariance models for iron-responsive elements. RNA Biology 8: 792–801. 21881407 10.4161/rna.8.5.16037

[15] Jacobs GH, Chen A, Stevens SG, Stockwell PA, Black MA, et al (2009) Transterm: a database to aid the analysis of regulatory sequences in mRNAs. Nucleic Acids Res 37: D72–76. 18984623 10.1093/nar/gkn763 PMC2686486

[16] Szostak E, Gebauer F (2012) Translational control by 3'-UTR-binding proteins. Brief Funct Genomics. 10.1093/bfgp/els056 PMC3548161 23196851

[17] Guo H, Ingolia NT, Weissman JS, Bartel DP (2010) Mammalian microRNAs predominantly act to decrease target mRNA levels. Nature 466: 835–840. 20703300 10.1038/nature09267 PMC2990499

[18] Lundberg E, Fagerberg L, Klevebring D, Matic I, Geiger T, et al (2010) Defining the transcriptome and proteome in three functionally different human cell lines. Mol Syst Biol 6: 450. 21179022 10.1038/msb.2010.106 PMC3018165

[19] Nagaraj N, Wisniewski JR, Geiger T, Cox J, Kircher M, et al (2011) Deep proteome and transcriptome mapping of a human cancer cell line. Mol Syst Biol 7: 548. 22068331 10.1038/msb.2011.81 PMC3261714

[20] Schwanhausser B, Gossen M, Dittmar G, Selbach M (2009) Global analysis of cellular protein translation by pulsed SILAC. Proteomics 9: 205–209. 19053139 10.1002/pmic.200800275

[21] Ong SE, Blagoev B, Kratchmarova I, Kristensen DB, Steen H, et al (2002) Stable isotope labeling by amino acids in cell culture, SILAC, as a simple and accurate approach to expression proteomics. Mol Cell Proteomics 1: 376–386. 12118079 10.1074/mcp.m200025-mcp200

[22] Cambridge SB, Gnad F, Nguyen C, Bermejo JL, Kruger M, et al (2011) Systems-wide proteomic analysis in mammalian cells reveals conserved, functional protein turnover. J Proteome Res 10: 5275–5284. 22050367 10.1021/pr101183k

[23] Vogel C, Abreu RD, Ko DJ, Le SY, Shapiro BA, et al. (2010) Sequence signatures and mRNA concentration can explain two-thirds of protein abundance variation in a human cell line. Mol Syst Biol 6. 10.1038/msb.2010.59 PMC2947365 20739923

[24] Meyuhas O (2000) Synthesis of the translational apparatus is regulated at the translational level. European journal of biochemistry/FEBS 267: 6321–6330. 10.1046/j.1432-1327.2000.01719.x 11029573

[25] Araujo PR, Yoon K, Ko D, Smith AD, Qiao M, et al (2012) Before It Gets Started: Regulating Translation at the 5′ UTR. Comp Funct Genomics 2012: 475731. 22693426 10.1155/2012/475731 PMC3368165

[26] Hinkson IV, Elias JE (2011) The dynamic state of protein turnover: It's about time. Trends Cell Biol 21: 293–303. 21474317 10.1016/j.tcb.2011.02.002

[27] Huang T, Shi XH, Wang P, He Z, Feng KY, et al (2010) Analysis and prediction of the metabolic stability of proteins based on their sequential features, subcellular locations and interaction networks. PloS one 5: e10972. 20532046 10.1371/journal.pone.0010972 PMC2881046

[28] Song X, Zhou T, Jia H, Guo X, Zhang X, et al (2011) SProtP: a web server to recognize those short-lived proteins based on sequence-derived features in human cells. PLoS One 6: e27836. 22114707 10.1371/journal.pone.0027836 PMC3218052

[29] Law GL, Bickel KS, MacKay VL, Morris DR (2005) The undertranslated transcriptome reveals widespread translational silencing by alternative 5′ transcript leaders. Genome Biol 6: R111. 16420678 10.1186/gb-2005-6-13-r111 PMC1414110

[30] Dressaire C, Gitton C, Loubiere P, Monnet V, Queinnec I, et al (2009) Transcriptome and proteome exploration to model translation efficiency and protein stability in Lactococcus lactis. PLoS Comp Biol 5: e1000606. 10.1371/journal.pcbi.1000606 PMC2787624 20019804

[31] Radonic A, Thulke S, Mackay IM, Landt O, Siegert W, et al (2004) Guideline to reference gene selection for quantitative real-time PCR. Biochem Biophys Res Commun 313: 856–862. 14706621 10.1016/j.bbrc.2003.11.177

[32] Andersen CL, Jensen JL, Orntoft TF (2004) Normalization of real-time quantitative reverse transcription-PCR data: a model-based variance estimation approach to identify genes suited for normalization, applied to bladder and colon cancer data sets. Cancer Res 64: 5245–5250. 15289330 10.1158/0008-5472.CAN-04-0496

[33] Tunbridge EM, Eastwood SL, Harrison PJ (2011) Changed relative to what? Housekeeping genes and normalization strategies in human brain gene expression studies. Biol Psychiatry 69: 173–179. 20673871 10.1016/j.biopsych.2010.05.023

[34] Thellin O, ElMoualij B, Heinen E, Zorzi W (2009) A decade of improvements in quantification of gene expression and internal standard selection. Biotechnol Adv 27: 323–333. 19472509 10.1016/j.biotechadv.2009.01.010

[35] Reich M, Liefeld T, Gould J, Lerner J, Tamayo P, et al (2006) GenePattern 2.0. Nat Genet 38: 500–501. 16642009 10.1038/ng0506-500

[36] Chepelev NL, Willmore WG (2011) Regulation of iron pathways in response to hypoxia. Free Radic Biol Med 50: 645–666. 21185934 10.1016/j.freeradbiomed.2010.12.023

[37] Bermano G, Shepherd RK, Zehner ZE, Hesketh JE (2001) Perinuclear mRNA localisation by vimentin 3'-untranslated region requires a 100 nucleotide sequence and intermediate filaments. FEBS Lett 497: 77–81. 11377416 10.1016/s0014-5793(01)02438-3

[38] Kleffmann T, von Zychlinski A, Russenberger D, Hirsch-Hoffmann M, Gehrig P, et al (2007) Proteome dynamics during plastid differentiation in rice. Plant Physiol 143: 912–923. 17189339 10.1104/pp.106.090738 PMC1803725

[39] Sury MD, Chen JX, Selbach M (2010) The SILAC fly allows for accurate protein quantification in vivo. Molecular & Cellular Proteomics 9: 2173–2183. 20525996 10.1074/mcp.M110.000323 PMC2953914

[40] Walther DM, Mann M (2011) Accurate quantification of more than 4000 mouse tissue proteins reveals minimal proteome changes during aging. Mol Cell Proteomics 10: M110 004523. 10.1074/mcp.M110.004523 PMC3033683 21048193

[41] Ghazalpour A, Bennett B, Petyuk VA, Orozco L, Hagopian R, et al (2011) Comparative analysis of proteome and transcriptome variation in mouse. PLoS Genet 7: e1001393. 21695224 10.1371/journal.pgen.1001393 PMC3111477

[42] Trapnell C, Pachter L, Salzberg SL (2009) TopHat: discovering splice junctions with RNA-Seq. Bioinformatics 25: 1105–1111. 19289445 10.1093/bioinformatics/btp120 PMC2672628

[43] Trapnell C, Williams BA, Pertea G, Mortazavi A, Kwan G, et al (2010) Transcript assembly and quantification by RNA-Seq reveals unannotated transcripts and isoform switching during cell differentiation. Nat Biotechnol 28: 511–515. 20436464 10.1038/nbt.1621 PMC3146043

[44] Wilkins MR, Gasteiger E, Bairoch A, Sanchez JC, Williams KL, et al (1999) Protein identification and analysis tools in the ExPASy server. Methods Mol Biol 112: 531–552. 10027275 10.1385/1-59259-584-7:531

[45] Smyth GK, Speed T (2003) Normalization of cDNA microarray data. Methods 31: 265–273. 14597310 10.1016/s1046-2023(03)00155-5

[46] Huang da W, Sherman BT, Lempicki RA (2009) Systematic and integrative analysis of large gene lists using DAVID bioinformatics resources. Nature protocols 4: 44–57. 19131956 10.1038/nprot.2008.211

