# Supplementary figures and images for: In Silico Estimation of Translation Efficiency in Human Cell Lines: Potential Evidence for Widespread Translational Control
Source: PLoS One. 2013 Feb 27;8(2):e57625. doi: 10.1371/journal.pone.0057625 (PMC3584024; doi:10.1371/journal.pone.0057625)

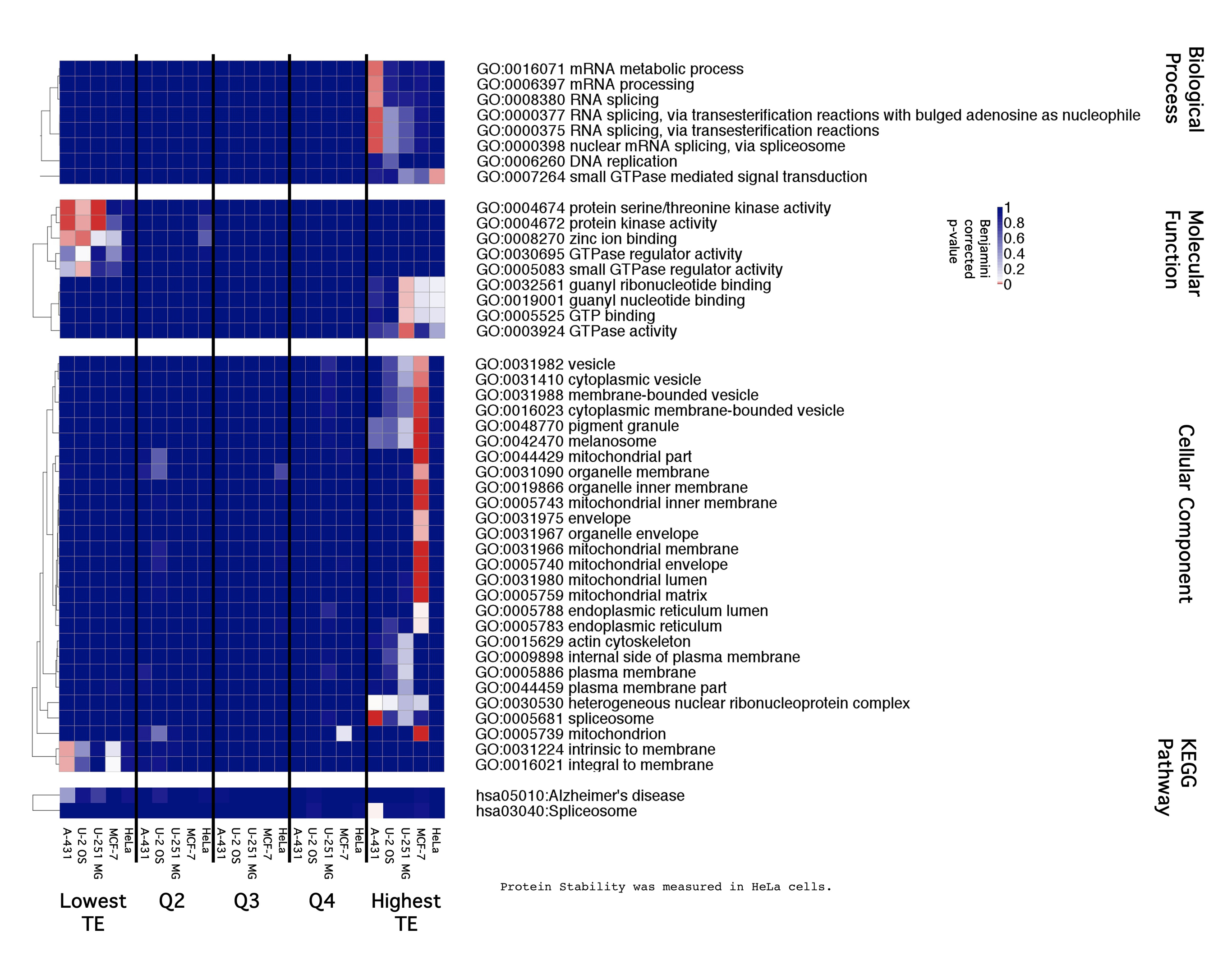

Supplement: Figure S2 — Functional characteristics of genes grouped by estimated translation efficiency calculated from HeLa protein stability data. Analysis of enrichment within gene ontology (GO) and KEGG pathway classifications for genes within each quintile of TE for each cell line. All ontologies shown have at least one enrichment passing a Benjamini corrected p-value<0.05. The colours in the figure correspond to corrected p-values such that colours from white to red show significance up to a significant p-value<0.05 (red). Only genes with protein stability data available in HeLa and NIH-3T3 cells are considered in the analysis. (TIF) [file pone.0057625.s002.tif]

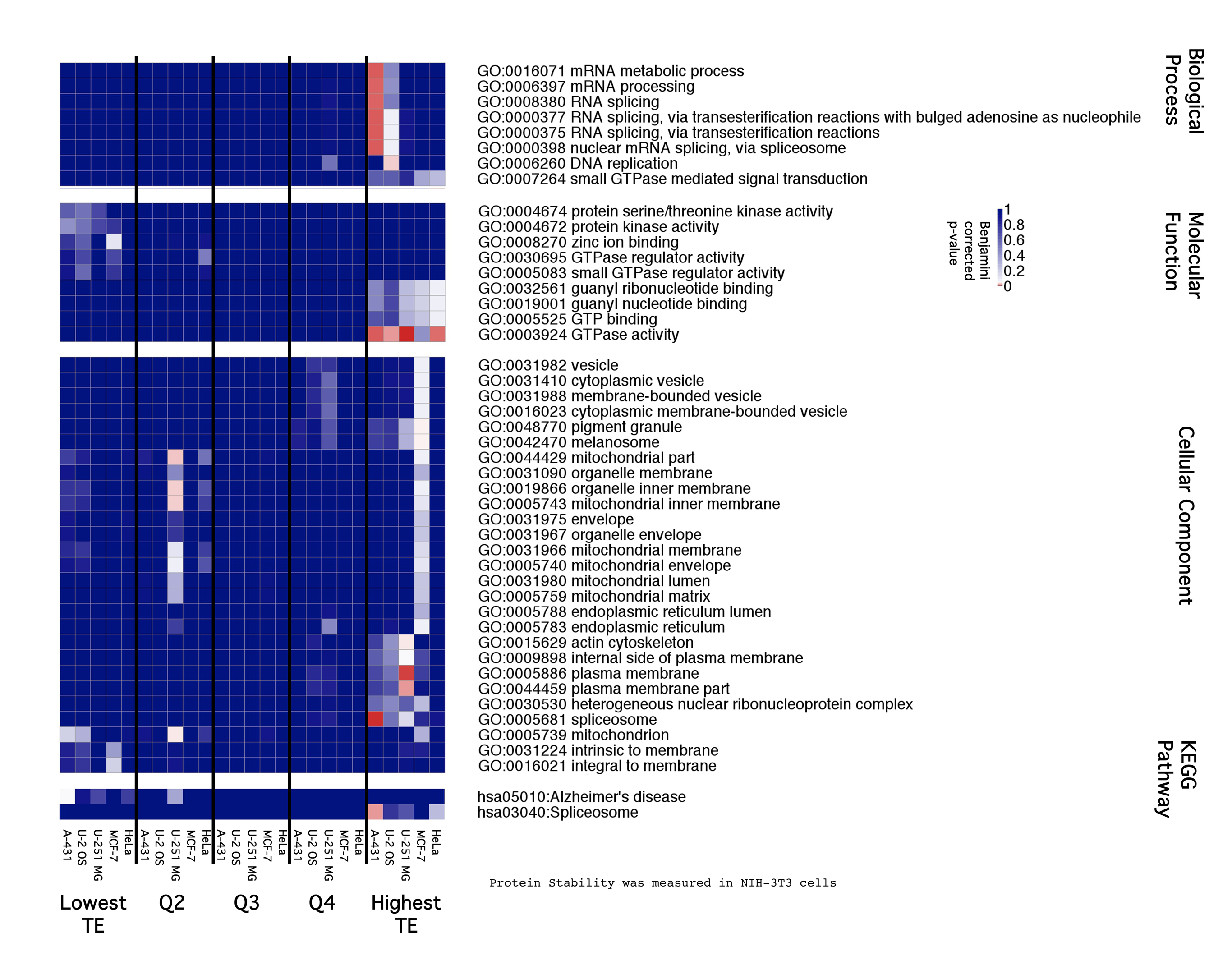

Supplement: Figure S3 — Functional characteristics of genes grouped by estimated translation efficiency calculated from NIH-3T3 protein stability data. Analysis of enrichment within gene ontology (GO) and KEGG pathway classifications for genes within each quintile of TE for each cell line. All ontologies shown have at least one enrichment passing a Benjamini corrected p-value<0.05. The colours in the figure correspond to corrected p-values such that colours from white to red show significance up to a significant p-value<0.05 (red). Only genes with protein stability data available in HeLa and NIH-3T3 cells are considered in the analysis. (TIF) [file pone.0057625.s003.tif]

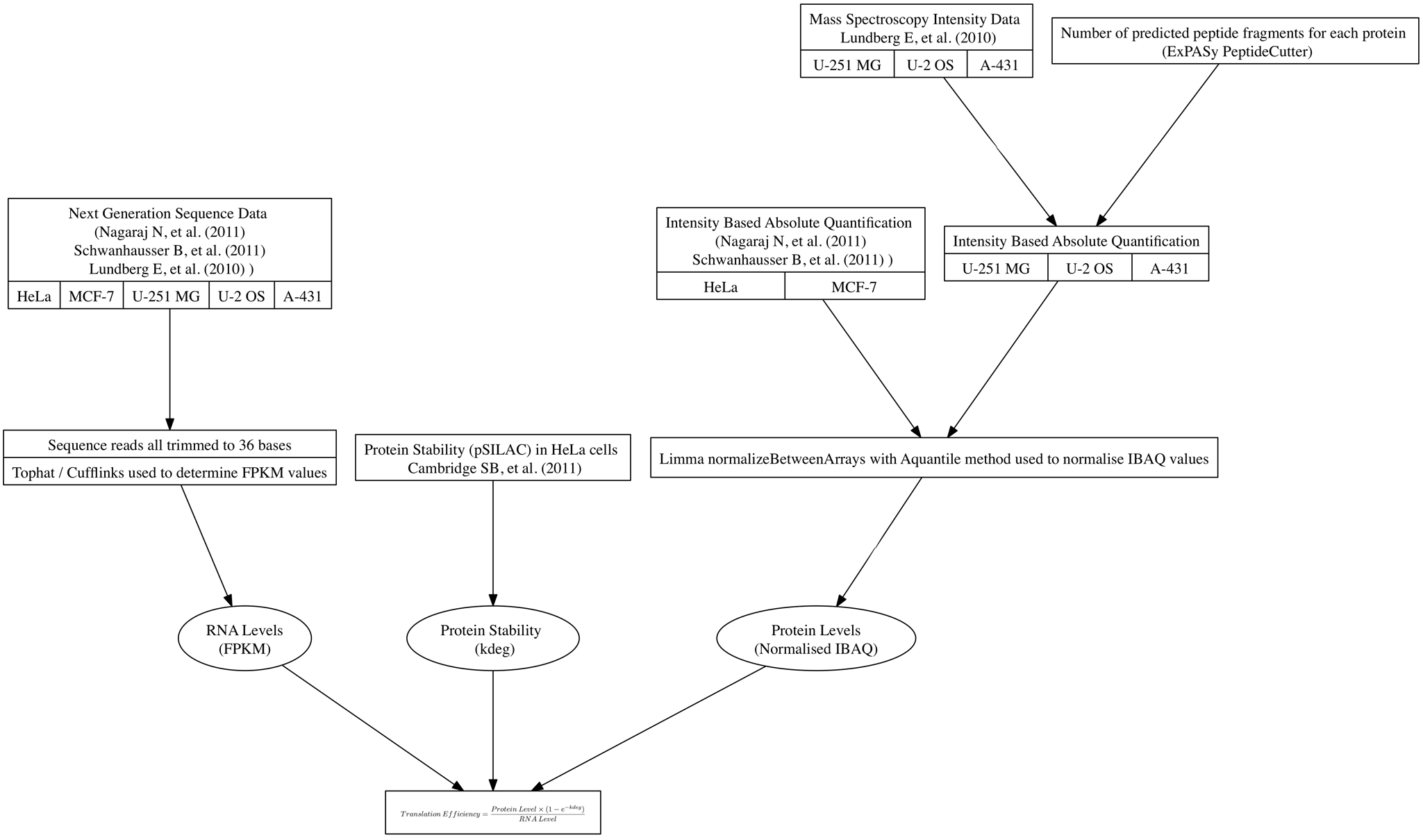

Supplement: Figure S4 — The pipeline used to estimate translation efficiency in the five cell lines. (TIF) [file pone.0057625.s004.tif]
