# Supplementary material for: In Silico Estimation of Translation Efficiency in Human Cell Lines: Potential Evidence for Widespread Translational Control
Source: PLoS One. 2013 Feb 27;8(2):e57625. doi: 10.1371/journal.pone.0057625 (PMC3584024; doi:10.1371/journal.pone.0057625)
Supplement: Material S1 — (DOC) [file pone.0057625.s007.doc]

***In silico* estimation of translation efficiency in human cell lines: potential evidence for widespread translational control.**

Stewart G Stevens^1^ and Chris M Brown^1,2^

^1^ Biochemistry and Genetics Otago, University of Otago, Dunedin, New Zealand.

^2^ Corresponding Author

**Supplemental Material**

In order to provide a gene by gene indication for the robustness of applying HeLa protein stability to the other four human cell lines, a comparison was made between normalised stability values from mouse fibroblast NIH-3T3 and human cervical cancer HeLa cell lines. For the 4,004 proteins with stability values available in HeLa cells there are 3,046 proteins with values also available from NIH-3T3 cells. Most (89%) had values within one standard deviation (see speadsheet). However, there is a difference in normalised protein stability of more than that for 11% (330) of these 3,046 genes. These 330 genes have ontology enrichment for cell cycle phase (Benjamani corrected p=0.032) and microtubule cytoskeleton organisation (Benjamani corrected p=0.014). This result may be indicative of disrupted proteolytic pathways effecting cell cycle, or different needs for microtubules in the two cell types.

We performed an enrichment analysis similar to that in Figure 3B, for those genes where both NIH-3T3 and HeLa data were available. Figure S2 shows such an enrichment analysis calculated using HeLa protein stability data – this is the same analysis as Figure 3 but using those ~75% of the total genes with protein stability data available for comparison. Whereas, for comparison, Figure S3 shows the enrichment analysis with the TE calculated using NIH-3T3 protein stability data.

In the analyses shown in Figures S2 and S3 the “steroid biosynthetic process”, “Pathways in Cancer”, “Ribosome” and several other terms lost enrichment in both. This was due to the availability of the protein stability data, for example, in the “ribosomal subunit” category 16 of the 36 genes showing enrichment had no protein stability value in NIH-3T3 cells, so do not feature in Figures S2 and S3. In addition, the “protein kinase activity”, “zinc ion binding” and “intrinsic to membrane” terms did not have consistent enrichments when the NIH-3T3 stability data was substituted. In these cases the estimate of TE was affected by cell specific regulation of protein stability. The “mRNA metabolic process”, “GTP binding”, “pigment granule”, “melanosome” and “Splicesome” terms all show consistent enrichments across the analyses.

There was a good protein by protein correlation (r=0.58), however the mean protein stability reported for the mouse NIH-3T3 cell line is about twice that for the HeLa cell line (half life of ~40 h vs. ~80 h).
